# Impacts of a Comprehensive Dementia Care Management Program on Patient and Caregiver Health and Wellbeing

**DOI:** 10.1002/alz.089924

**Published:** 2025-01-09

**Authors:** Zaldy S Tan, Nabeel Qureshi, Erica Spivack, Deana Rhinehart, Dyane Gatmaitan, Augustine Guinto, Sarah Kremen, Nancy Sicotte

**Affiliations:** ^1^ Cedars‐Sinai Medical Center, Los Angeles, CA USA

## Abstract

**Background:**

The United States faces a growing challenge with over 6.5 million people living with dementia (PLwD). PLwD and their caregivers struggle with cognitive, functional, behavioral, and psychosocial issues. As dementia care shifts to home settings, caregivers bear increasing responsibilities, leading to increase strain. This study assesses the initial impacts of a pragmatic dementia care model, the Cedars‐Sinai C.A.R.E.S. Program, on patient and caregiver health and wellbeing.

**Methods:**

The Cedars‐Sinai C.A.R.E.S. Program is an integrated care management program that enrolled eligible patients aged 65 and older with a dementia diagnosis or dementia medication within the health system. Enrolled patients underwent comprehensive assessments of patient and caregiver health and wellbeing using the Neuropsychiatric Inventory‐Questionnaire (NPI‐Q) (patient), Patient Health Questionnaire (PHQ) 2 and 9 (caregiver), and Modified Caregiver Strain Index (MCSI) (caregiver) at baseline and follow up one‐year after enrollment. We calculated overall and sub‐assessment scores for each measure and compared the scores using t‐tests to test for statistical significance.

**Results:**

To date, of the 431 were enrolled in the C.A.R.E.S. Program, 80 completed their baseline and follow‐up health and wellbeing assessment. Assessment scores were higher (worse health and lower wellbeing) at baseline than at follow up for all measures. For the NPI‐Q, the overall score decreased from 23.54 (SD = 15.68) to 20.74 (17.71) (p = 0.29), the distress sub‐assessment score decreased from 10.7 (7.94) to 9.42 (8.85) (p = 0.34), and the severity sub‐assessment score decreased from 8.3 (5.6) to 7.36 (6.48) (p = 0.33), though none were statistically significant. Similarly, caregiver depression decreased from 1.12 (1.41) to 0.8 (1.19) using the PHQ‐2 (p‐0.13) and decreased from 4.73 (5.32) to 4.22 (4.61) using the PHQ‐9 (0.52). Finally, caregiver strain decreased from 9.84 (6.25) to 8.47 (5.84) (p = 0.15).

**Conclusions:**

The Cedars‐Sinai C.A.R.E.S. Program offers a promising approach to dementia care. While there were differences in assessment scores at baseline and follow‐up, differences were not statistically significant. As the program continues to enroll patients and they complete their assessments, we expect the increase in statistical power will help determine whether differences are real or due to chance.

